# Low bioavailability of dietary iron among Brazilian children: Study in a representative sample from the Northeast, Southeast, and South regions

**DOI:** 10.3389/fpubh.2023.1122363

**Published:** 2023-02-20

**Authors:** Carlos Alberto Nogueira-de-Almeida, Daniela Prozorovscaia, Elaine M. Bento Mosquera, Fábio da Veiga Ued, Vanessa Caroline Campos

**Affiliations:** ^1^Medical Department, Federal University of São Carlos, São Carlos, Brazil; ^2^Nestlé Institute of Health Sciences, Nestlé Research, Lausanne, Switzerland; ^3^Federal University of São Paulo, São Paulo, Brazil; ^4^Medical, Scientific and Regulatory Affairs - Nestlé Nutrition/Nestlé Brazil Ltda, São Paulo, Brazil; ^5^FMRP - Nutrition Department, University of São Paulo, São Paulo, São Paulo, Brazil

**Keywords:** anemia, iron deficiency, bioavailability, ascorbic acid, chelating agents, Brazil

## Abstract

**Background:**

Despite all efforts, iron deficiency anemia remains a serious public health problem among Brazilian children.

**Objective:**

To evaluate dietary iron intake and dietary practices that interfere with the absorption of this nutrient from three regions of Brazil.

**Methods:**

Brazil Kids Nutrition and Health Study is a cross-sectional dietary intake study in children aged 4–13.9 years old designed to investigate nutrient intakes and gaps of Brazilian children in a representative sample of households from Northeast, Southeast and South regions. Nutrient intake was assessed based on multiple-pass 24-h dietary recall and U.S. National Cancer Institute method was used to estimate usual micronutrients intakes and compliance with Dietary Reference Intakes.

**Results:**

Five hundred sixteen individuals participated in the study (52.3% male). The top three most consumed food sources of iron were products of plant origin. Food sources of animal origin contributed with <20% of the total iron intake. Vitamin C intake was adequate, but the concomitant consumption of food sources of vitamin C with plant food sources of iron was not common. On the other hand, the concomitant intake of plant food sources of iron with food sources of iron chelators (e.g., coffee and teas) was frequent.

**Conclusions:**

Adequate iron intake was observed in all three regions in Brazil. Children's diet showed low iron bioavailability and insufficient consumption of food sources of iron absorption stimulants. Frequent presence of iron chelators and inhibitors of iron absorption might help to explain the high prevalence of iron deficiency in the country.

## Introduction

Anemia is a serious global public health problem particularly affecting young children and pregnant women (1). In Brazil, a recent nationwide meta-analysis observed a prevalence of anemia of 38, 36, 35, and 28% in the Northeast, Midwest, South and Southeast regions of Brazil, respectively, in children from 0 to 7 years old (2). Data obtained by the ENANI study showed prevalences of 10% for the country as a whole, but reaching 17% in the North and 19 % for children under 2 years of age (3). As worldwide (4), the most common cause of anemia in Brazil is iron deficiency mainly due a low consumption of dietary iron (3, 5–8). Since iron plays a fundamental role in child development, especially in the development of the central nervous system (9), iron deficiency anemia leads to important health issues as poor motor and cognitive development, growth problems and impaired immune system (10–12). There are several consequences of iron deficiency anemia in childhood, highlighting negative interference in the production and action of cytokines, in the phagocytic capacity of neutrophils and macrophages (13) and in the production of T lymphocytes (14), leading to lower immunity (15, 16), psychomotor changes (17), impairment of thyroid function at risk of affecting growth (18–21) and unsatisfactory cognitive development (9) with learning impairment (17, 22–25).

Understanding the causes underlying iron deficiency anemia is crucial and some of them have been listed to explain the high prevalence in Brazil: use of unmodified cow's milk after weaning (26) associated with the low use of fortified products in complementary feeding (27); low reserve accumulated by infants during pregnancy and lactation due to the high prevalence of iron deficiency among pregnant and lactating women (28); low bioavailability of dietary iron due to reduced intake of meat and fortified products (29); presence of iron sources mainly of plant origin, with low bioavailability, in addition to the abundance of phytates and other chelators (30, 31).

Brazilian publications evaluating food intake and nutritional factors that interfere with iron absorption in some age groups are rare, but necessary to understand the high rates of anemia in Brazilian children. For this reason, the present study aimed to evaluate dietary iron intake and dietary practices that interfere with the absorption of this nutrient in children aged 4–8 years old from three regions of Brazil.

## Methods

### Study design and population

The Brazil Kids Nutrition and Health Study (B-KNHS) is a cross-sectional dietary intake study in children aged 4–13.9 years old (*n* = 983) designed to investigate the nutrient intakes and gaps of Brazilian children in a representative sample of the three main geographic regions in Brazil (Northeast, Southeast, and South), among urban and rural households. Detailed methodology information of B-KNHS were published at a previous paper (32). The study was designed to obtain a representative sample within the three regions of interest. The sample size included four stages of selection (geographic region, state, sector, and household) to ensure maximum randomization. First, we stratified the population into nine geographical regions composed of groupings of states. We sampled a total of nine states, with at least one state in each region: Northeast, Southeast, and South. Next, population sectors were selected in proportion to the estimated the appropriate number of children. Population estimates were based on the 2008 National Household Sample Survey (PNAD), conducted by the Brazilian Institute of Geography and Statistics (IBGE). Within each selected sector, households were randomly recruited at a starting point and then field interviewers conducted a random walk through the sector, adhering to a set of rules that specified household selection according to the estimated number of children within the sector. Within eligible households, one child at the age range was randomly selected. Data from 516 children, restricting the age group to those aged between 4 and 8.9 years old, were analyzed: Northeast (*n* = 169), South (*n* = 162) and Southeast (*n* = 185). The number of subjects was proportional to the population in each region, with a study response rate of 70.4%. Regarding the 29.6% that did not agree to respond, we chose, for ethical reasons, not to ask why and these families were not included in the analyses.

The survey protocol and data collection instruments were approved by the institutional review boards of RTI International, *Faculdade de Ciências Farmacêuticas* from University of São Paulo and the National Commission for Research Ethics (*Comissão Nacional de Ética em Pesquisa*). Informed consent was obtained from each child's parent/guardian for participation in the study.

### Data collection

In-person interviews were conducted with each child and caregiver in their house from September to December 2019. A structured questionnaire was used to collect information on socio-demographic characteristics of households and caregivers, child characteristics including anthropometric measurements, lifestyle parameters, dietary patterns, and food intakes. The Brazilian Economic Classification Criteria were used for the economic stratification of the population. The questionnaire for family economic status covered parents' schooling and the presence/absence and number of domestic appliances, vehicles, and rooms in the child's home. Families were classified into categories from A (highest) to E (lowest). All interviewers were trained on study procedures and interviewing techniques *via* standard protocol which was tested during a pilot study run with 60 subjects.

### Dietary assessment

Nutrient intake was assessed by trained interviewers based on multiple-pass 24-h dietary recall to capture a detailed list of foods and beverages consumed by each child. Information included the quantity consumed, preparation method (e.g., boiled, fried, added sugar, salt, etc.); eating occasion (e.g., breakfast, lunch, dinner, snacks); and eating location (e.g., home, someone else's home, school, daycare, restaurant, party/event, traveling or other). A second 24-h recall was collected from a random 25% subsample to estimate within-person variance for estimating usual nutrient intakes. Parents or primary caregivers responded on behalf of children under 6 years of age. Primary caregivers were interviewed in the presence of children 6–8 years, so children could provide additional information, such as foods eaten at school. A booklet adapted for the study including food measurements guide was used to support interviewers in recording detailed information on type of food consumed and the quantity in household measurements. Intake data were entered into Nutrition Data System for Research software (NDSR, version 2018, University of Minnesota, Minneapolis, MN) to estimate energy and nutrient content of foods and beverages consumed. The data base was completed with local foods and recipes. Foods added were typically foods identified several times in the interviews or foods commonly consumed in a particular region that were not yet in the database.

### Statistical analysis

All statistical analyses were performed with the use of SAS (version 9, SAS Institute Inc.) and SAS-callable SUDAAN^®^ (version 11, RTI International) software. The U.S. National Cancer Institute method (33) was used to estimate usual micronutrients intakes and compliance with Dietary Reference Intakes (DRIs) assessing the probabilities of meeting the Estimated Average Requirement (EAR, % < EAR) and exceeding the Adequate Intake (AI, % >AI) or Tolerable Upper Intake Level (UL, % >UL). To investigate the food sources of nutrients, a food classification system was designed to group foods based on major and minor food categories. This system was based on the food classification system used in previous dietary intakes studies in young children in the USA (34), adjusted to incorporate local food culture of Brazilian children. The percentage of children consuming foods and food groups on a given day (%), amount consumed (g) and percent contribution to total energy intake (TEI) were calculated.

## Results

A total of 516 individuals participated in the study (52.3% male). The distribution in social classes respects the proportion found in Brazil, in which the majority of the population is in the middle class (C1 and C2), followed by the lower classes (D and E) and a minority in the upper classes (A and B) (35). Almost 80% of the main caregivers have intermediate education, between elementary and high school. Most families have an income of more than $500 (equivalent to ~2.3 local minimum wages), and about 1/3 reported receiving financial support from the government to buy food (Table 1).

**Table 1:** Demographic characteristics of Brazilian children aged 4–8 years living in the Northeast, Southeast, and South regions of the country.

	**Overall**	**Northeast**	**Southeast**	**South**
	***n* (%)**	***n* (%)**	***n* (%)**	***n* (%)**
Population	516 (100.0)	169 (32.8)	185 (35.9)	162 (31.4)
**Gender**				
Boys	270 (52.3)	89 (33.0)	97 (35.9)	84 (31.1)
Girls	246 (47.7)	80 (32.5)	88 (35.8)	78 (31.7)
**Socioeconomic status**				
D, E	157 (30.4)	86 (54.8)	46 (29.3)	25 (15.9)
C1 + C2	267 (51.7)	72 (27.0)	90 (33.7)	105 (39.3)
A, B	92 (17.8)	11 (12.0)	49 (53.3)	32 (34.8)
**Caregiver education**				
No/incomplete school	39 (7.6)	22 (56.4)	7 (17.9)	10 (25.6)
Elementary	98 (19.0)	43 (43.9)	26 (26.5)	29 (29.6)
Jr. High school	117 (22.7)	37 (31.6)	42 (35.8)	38 (32.5)
High school	199 (38.6)	59 (29.6)	81 (40.7)	59 (29.6)
Higher education	39 (7.6)	8 (20.5)	20 (51.3)	11 (28.2)
No response	24 (4.7)	NA	NA	NA
**Additional children in household**				
0	201 (39.0)	64 (31.8)	71 (35.3)	66 (32.8)
1	203 (39.3)	65 (32.0)	73 (36.0)	65 (32.0)
2	72 (14.0)	25 (34.7)	27 (37.5)	20 (27.8)
3	28 (5.4)	12 (42.9)	10 (35.7)	6 (21.4)
4 or more	12 (2.3)	3 (25.0)	4 (33.3)	5 (41.7)
Receive and use government support	193 (37.4)	105 (54.4)	56 (29.0)	32 (16.6)
**Household income**				
Less than $477	28 (5.4)	25 (89.3)	3 (10.7)	0 (0.0)
477–954	83 (16.1)	44 (53.0)	27 (32.5)	12 (14.5)
955–1,908	187 (36.2)	73 (39.0)	72 (38.5)	42 (22.5)
1,909–2,862	115 (22.3)	20 (17.4)	40 (34.8)	55 (47.8)
2,863–4,770	65 (12.6)	4 (6.2)	25 (38.5)	36 (55.4)
4,771–9,540	20 (3.9)	1 (5.0)	7 (35.0)	12 (60.0)
9,541+	6 (1.2)	0 (0.0)	4 (66.7)	2 (33.3)
No answer	12 (2.3)	NA	NA	NA

NA, not applicable.

The inadequacy of micronutrient intake is shown in Table 2. The percentage of inadequate intake of magnesium, zinc, iron, phosphorus, niacin and folate was below 2%. Vitamins C (8%) and A (20%) also had low values of inadequate intake (Table 2). However, vitamins E, D, K and choline, as well as potassium and calcium had percentages of inadequacy above 67%. Although there is variability in the inadequacy of intake between different nutrients, higher prevalence of inadequate intakes was found among children of lower classes and for those from Northeast region.

**Table 2:** Prevalence of inadequate intake of micronutrients by Brazilian children aged 4–8 years in the Northeast, Southeast, and South regions and according to socioeconomic status.

**% of children ingesting nutrients below EAR recommendations**							
	**Overall**	**By socioeconomic status**			**By region**		
		**D, E**	**C1** + **C2**	**A, B**	**Northeast**	**Southeast**	**South**
Vitamin C	8.0	22.0	0.9	3.0	6.5	3.8	20.0
Magnesium	1.5	1.0	2.0	0.0	0.0	2.0	1.8
Zinc	0.0	0.0	0.0	0.0	0.0	0.1	0.0
Iron	0.1	0.0	0.0	0.0	0.0	1.0	0.0
Phosphorus	0.1	0.0	0.0	0.0	0.0	0.0	0.0
Vitamin A	20.0	28.0	28.0	0.0	26.0	1.0	37.0
Vitamin E	78.0	86.0	74.0	79.0	83.0	78.0	68.0
Calcium	80.0	85.0	79.0	74.0	82.0	87.0	69.0
Vitamin D	100.0	100.0	100.0	100.0	100.0	100.0	100.0
Vitamin K	72.0	78.0	70.0	63.0	95.0	66.0	52.0
Potassium	91.0	93.0	90.0	98.0	98.0	92.0	80.0
Choline	67.0	70.0	68.0	16.0	100.0	67.0	58.0
Niacin	1.5	2.8	0.8	0.0	0.1	0.5	0.0
Folate	0.2	0.0	0.8	0.0	0.0	0.3	0.5

Regarding the consumption of food sources of iron (Table 3), for all regions, the first three most consumed food sources were products of plant origin, such as pulses, breads and sweet bakery products, which contributed with 38% of the iron daily ingested. Food sources of animal origin appeared from the fourth position and contributed with < 20% of the total iron intake, in the different regions, considering all meat and meat products together.

**Table 3:** Main dietary sources of iron consumed by Brazilian children aged 4–8 years in the Northeast, Southeast, and South regions of the country.

**Overall**		**Northeast**		**Southeast**		**South**	
**Foods**	**Contribution to daily iron intake (%)**	**Foods**	**Contribution to daily iron intake (%)**	**Foods**	**Contribution to daily iron intakes (%)**	**Foods**	**Contribution to daily iron intakes (%)**
Pulses	13	Sweet bakery	13	Rolls	16	Pulses	16
Rolls	13	Rolls	11	Pulses	16	Sweet bakery	12
Sweet bakery	12	Pulses	10	Sweet bakery	11	Rolls	11
Beef	8	Wholegrains	10	Beef	8	Beef	10
Whole grains	6	Beefs	8	Chicken/turkey	5	Bread	5
Chicken/turkey	5	Porridge	7	Rice	4	Spaghetti, ravioli, lasagna	5
Baby cereal	4	Breakfast cereals	7	Spagetti, ravioli, lasagna	3	Chicken/turkey	5
Rice	4	Chicken/turkey	5	Sweetened beverages	3	Rice	5
Breakfast cereal	4	Cow's milk	5	Wholegrains	3	Soups	4
Cow's milk	4	Crackers, pretzels, rice cakes	4	Porridge	3	Cow's milk	3
Sweetened beverages	3	Rice	4	Soups	3	Sweet beverages	2
Crackers, pretzels, rice cakes	3	Sweet beverages	4	Crackers, pretzels, rice cakes	2	Fruits	2
Spagetti, ravioli, lasagna	3	Soups	2	Cow's milk	2	Hot dog, cold cuts, sausage, bacon	2
Soups	3	Eggs/egg dishes	2	Bread	2	Wholegrains	2
Bread	2	Beef with vegetables and/or rice or pasta	2	Chips, other salty snacks	2	Vegetables	2
Chips and other salty snacks	2	Fruits	1	Hot dog, cold cuts, sausage, bacon	2	Breakfast cereals	2
Fruits	2	Spaghetti, ravioli, lasagna	1	Breakfast cereals	2	Sandwich	2
Eggs/egg dishes	2	Chips, other salty snacks	1	Eggs/egg dishes	2	Chips, other salty snacks	1
Hot dogs, cold cuts, sausages, bacon	1	Vegetables	1	Fruits	2	Crackers, pretzels, rice cakes	1
Vegetables	1	Bread	1	Vegetables	1	Baby cereals, porridge	1
Beef with vegetables	1	Sandwich	1	Pizza	1	Eggs /egg dishes	1
Pizza	1	Hot dog, cold cuts, sausage, bacon	1	Organ meats	1	Pizza	1
Sandwich	1	Pork/ham	1	Pasta	1	Beef with vegetables and/or rice or pasta	1
Organ meats	1	Organ meats	1	White potatoes	1	White potatoes	1
Pasta	1	Fish and shellfish	1	Beef with vegetables and/or rice and/or pasta	1	Ice cream, frozen yogurt, puddings	1

**Figure 1:**
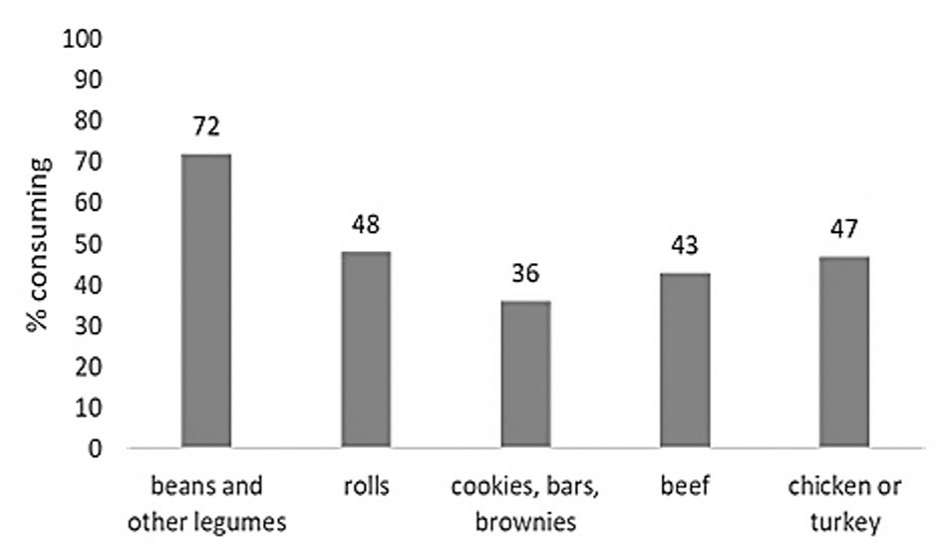
Percentage of Brazilian children aged 4–8 years who consume the five main dietary sources of iron.

**Figure 2:**
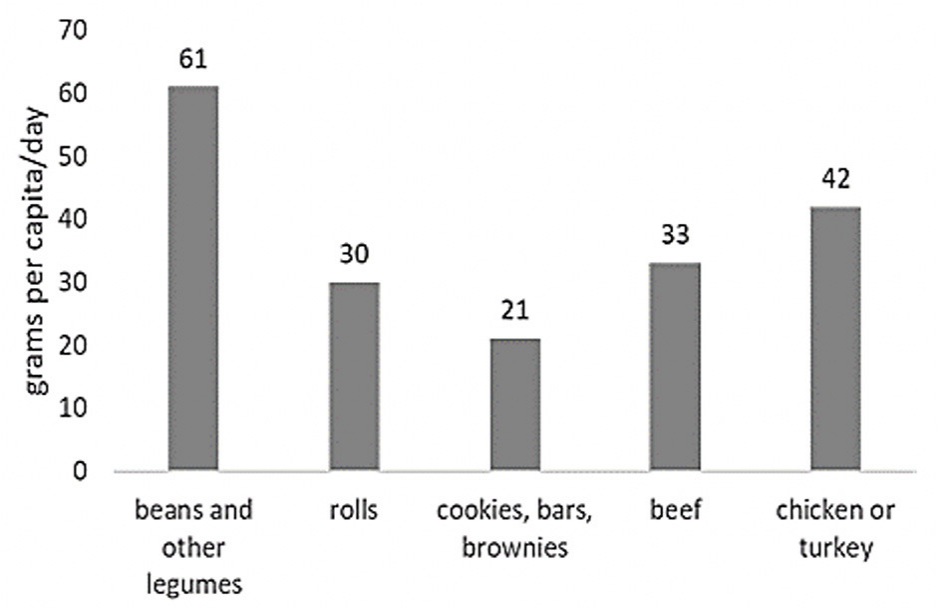
Per capita daily intake (g/day) of the five most relevant sources of iron in the diet of Brazilian children aged 4–8 years old.

Almost half of Brazilian children did not consume animal food sources of iron such as beef and chicken/turkey (Figure 1). Figure 2 shows the daily per capita consumption of the five most relevant sources of iron for this sample, with 75 g/day for meat, in contrast to 112 g/day when adding up the other iron food sources. The main dietary sources of vitamin C, a stimulant of non-heme iron absorption, were artificial juices, fruits, vegetables, and 100% natural juices (Table 4).

**Table 4:** Main dietary sources of vitamin C consumed by Brazilian children aged 4–8 years in the Northeast, Southeast, and South regions of the country.

**Food sources of vitamin C**	**% of dietary intake**
Fruit-flavored drink	30.5
Fruits	14.9
Bananas	5.3
Other fruits	3.5
Apple	2.5
Orange/citrus	2.1
Mango	1.6
Pear	1.2
Vegetables	8.9
Orange and red vegetables	4.8
White potatoes	3.5
Other vegetables	1.8
Starchy vegetables	1.2
Dark green vegetables	1.1
100% Juice	8.5
Citrus/Citrus Blend	2.1
Milk flavorings	6.3
Spaghetti, ravioli, lasagna	3.7
Non-meat protein sources (excl cheese/yogurt)	3.3
Soup	2.8
Sweet bakery	2.6
Meats	2.4
Cow's milk	2.2
Cereal, Family (RTE or Hot)	1.7
Yogurt	1.4
Wholegrain	1.1
Baby food, cereal	1.1

Table 5 shows the concomitant presence of iron-rich plant foods and non-heme iron absorption stimulator/inhibitor substances in the three main daily meals (breakfast, lunch, and dinner). A breakfast, the five most consumed foods were milk, bakery products, tea, coffee, water, and milk flavoring, observing the concurrence of possible sources of iron *via* fortification (for example, milk and cereals) and inhibitors such as tea, coffee, and calcium. At lunch, rice, meat, breads, artificial juices, and water appear with a low prevalence of consumption of vegetable iron sources and vitamin C sources. At dinner there is a greater intake of meat, rice, water, beans, and desserts. Vegetable sources of iron are represented by beans (fourth position) associated with the consumption of vegetables and fruits that are sources of vitamin C present in the 10 first positions. However, it was also verified the presence of iron chelators in this meal, represented by tea and coffee.

**Table 5:** Main foods and beverages consumed by Brazilian children aged 4–8 years in the Northeast, Southeast and South regions, for breakfast, lunch, and dinner.

**Breakfast**		**Lunch**		**Dinner**	
**Foods**	**% Consumption**	**Foods**	**% Consumption**	**Foods**	**% Consumption**
Cow's milk	59	Rice	83	Meats	49
Bread, rolls, biscuits, tortilla	40	Meats	74	Rice	44
Tea and coffee	39	Pulses	68	Water	36
Water	21	Fruit flavored drinks	37	Beans	34
Milk flavorings	21	Water	36	Mixed dishes	21
Margarine	20	Vegetables orange and red	22	Fruit flavored drinks	17
Sweet bakery	15	Mixed dishes	21	Vegetables orange and red	10
Non-meat protein sources	15	Pasta	13	Cow's milk	10
Sugar, syrups, preserves, and jelly	11	Condiment and sauces	13	Tea and coffee	9,2
Whole grains	8.1	White potatoes	12	Eggs	9.1
Crackers, pretzels, rice cakes	8.1	Soft drinks	10	Soft drinks	8.8
Meats	8	Sugar, syrups, preserves, and jelly	7.8	Pasta	6.5
Porridge	5.4	Salad dressing	6.7	White potatoes	6.2
Fruit flavored drinks	5	Oil	6.6	Condiments and sauces	5.7
Butter	3.8	100% fruit juice	5.7	Bread, rolls, biscuits, tortilla	5.3
Banana	3.1	Vegetables dark green	2.8	Sugar, syrups, preserves, and jelly	5.2
Vegetables	2.5	Banana	1.4	Other vegetables	4.9
Apple	1.4	Melon	1.1	Salad dressing	3.8
100% fruit juice	1.4	Orange/citrus	0.7	Whole grains	3.3
Other fruit	1.2			100% fruit juices	3.3

## Discussion

The prevalence of anemia in Brazil has shown a decline in recent years. Data from two meta-analyses (36, 37), published at 12-year intervals, show that the prevalence of anemia dropped by 23% among children aged ≤ 5 years. Despite this decrease, the numbers are still off concern due to the important impact on health. Children in the first year of life, might be at risk of not developing the necessary iron reserves and therefore, require, for the subsequent years of life, a higher intake of foods rich in high bioavailable iron, while limiting the concomitant intake of food sources of iron chelators (38).

The objectives of this study were to learn about the iron intakes, food sources of iron and dietary practices which may interfere with iron absorption of diets consumed by Brazilian children in the three most populated regions of the country. It was verified that only 30%−40% of children consumed beef or chicken in the 24 h prior to the interview. In contrast, 72% reported to consume legumes. Suggesting that the main sources of iron are of plant origin which, in addition to being of low bioavailability, they also contain iron chelators, such as phytates (30, 31). Unmodified cow's milk might also contribute to this process, especially because of the low iron content of this food and its low bioavailability (39). Although milk is often referred to as an iron chelator due to its calcium content, this fact has been questioned in other studies (40). Evidence indicates that this interference only exists when there is a concomitant intake of iron and calcium and especially when calcium intake is in amounts above the daily recommendations (DRI's) (41).

The data of the present study shows similar results to others already published, demonstrating several inadequacies of micronutrients intake (42). By the other hand, the prevalence of iron inadequate ingestion is extremely low contrasting with the high rates of anemia. This suggests that the iron requirements are covered by dietary iron, however its bioavailability might be inadequate (38). The top three food sources of iron consumed by Brazilian children were of plant origin, for all regions, representing more than 1/3 of all iron ingested. On the other hand, meat contributed with < 20% of the total iron intake and more than half of children did not consume meat in the 24 h prior to the interview. Similar results have already been observed by other authors and it is hypothesized that the intake of iron *via* plant sources (low bioavailability), the low intake of iron absorption stimulants (which could increase the bioavailability of non-heme iron) and the low meat consumption (high iron bioavailability) contribute significantly for iron deficiency establishment (6, 8, 10, 27, 30–32, 43).

In the study by Grillo et al. the consumption of fruits was considered insufficient in 48% of the children and vegetables in 80% (44). In the present study, similar results were found. More than 60% of children aged 4–8 years did not consume vegetables (excluding potatoes) in the 24 h prior to the interview and 58% of children did not consume fruit or fruit juices. Additionally, in the present study, it was found that the prevalence of inadequacies was more severe in the lower classes and most children from low-income families were from the Northeast (Table 1). Study by Borges et al. found a prevalence of anemia of 20.27% in children aged 7–9.9 years in Salvador, northeastern Brazil, where poverty and food intake with low iron bioavailability were the most relevant explanatory factors (6). Data from Manaus, in the North region of the country, showed a prevalence of anemia of 23.8% among 122 schoolchildren aged between 6 and 10 years; this study showed a relationship of anemia with low socioeconomic status and consumption of foods of low nutritional value and poor in iron of good bioavailability (45).

In the present study, amongst the most consumed food sources of iron were pulses and flour-based products. In Brazil, the fortification of wheat and corn flours with iron and folic acid is mandatory since 2004 (46). This may contribute to the total iron intake being adequate. However, a low impact of this fortification on the prevalence of anemia among children has already been demonstrated, possibly due to the small amount of farinaceous ingested in this age group, associated with the low bioavailability of iron added by mills (47). In addition, the consumption of foods such as beans, highly present at lunch and dinner in this sample, may not be the most viable strategy of achieving the daily recommendations of iron, considering that such group of foods presents in its composition factors that impair the absorption of iron, such as phytic acid and oxalic acid (48). It is estimated that only about 17% of the iron contained in cooked beans is used, while 34% of the iron present in the beef is available to the body (48).

The concomitant use of foods capable of stimulating the absorption of non-heme iron, especially vitamin C, could improve the utilization of iron from plant sources and fortified foods (43, 49, 50). In this context, fruits, natural juices, and even other foods fortified with ascorbic acid can help if ingested in a time close to the consumption of non-heme iron (43, 49). da Silva Ferreira et al. (51) evaluated the dietary practices of children leaving in Maceió (northeastern region of Brazil) and it was found a relationship between the presence of anemia and low consumption (< 2 portions per day) of fruits or fruit juices (51). In the present study, even though vitamin C intake was not low, temporal coincidence between the intake of food sources of iron and food sources of vitamin C was not observed in the three main meals. On the other hand, the concomitance of iron chelators, such as coffee and teas (52), was frequent.

Fortifying frequently consumed foods, such as milk, with highly available iron, can be an effective alternative to decrease iron deficiency (53), especially when fortification is based on sources of highly bioavailable iron (i.e., sulfate) or in combination with vit C (43, 53, 54), FAO/WHO considers that food fortification can be a quick and effective way to improve nutrient deficiency rates in populations (12). Furthermore, FAO/WHO recommends using highly bioavailable forms that do not alter the organoleptic characteristics of the food, and reinforces the fortification of common foods in infant feeding, such as milk, citing international experiences such as Chile, in which iron fortification in milk resulted in a rapid reduction in the rates of mineral deficiency (12). The success of this type of initiative may be in the fact that, when consumed regularly and frequently, fortified foods are able to maintain body iron stock, which is an advantage when compared to other types of initiatives, such as supplementation, which is intermittent (55).

The current study has some limitations. Food consumption in the 24 h prior to the interview does not represent the eating habits of the study population. In addition, data were collected in three Brazilian regions (Northeast, Southeast and South), leaving out two regions (North and Midwest). Despite this, it is possible to extend the data found to the entire population of Brazilian children, due to the North and Midwest regions having a human development index (HDI) and per capita income similar to the Northeast region, and due to the representative sample collected in the other regions. Finally, the questionnaire applied did not include information on the use of supplements (vitamins and minerals) that are frequently used by children and this eventual use has the potential to modify the nutritional status of micronutrients.

In conclusion, adequate iron intake was observed in all three regions of Brazil. On the other hand, the composition of the children's diet showed that the top food sources of iron were of plant origin. Moreover, within the same meal, an insufficient consumption of iron absorption stimulants, such as vitamin C, and a frequent presence of iron chelators and inhibitors of iron absorption was observed. Considering that the nutritional status of iron depends not only on the amount ingested but also on the ability of the human body to utilize the consumed iron, since the main mechanism for maintaining iron homeostasis in the human body is its absorbed amount (56), it is recommended that the bioavailability of the mineral in question be considered, to optimize its use by the human body and ensure greater fortification effectiveness. Similarly, two other factors should be considered, especially when referring to the time of consumption of foods sources of iron: the presence of other foods whose nutrients may increase their absorption (such as vitamin C), as well as the presence of chelating agents, which may negatively impact this process. Furthermore, we suggest that current dietary recommendations also include the minimum intake of highly bioavailable iron.

It is necessary to consider the use of iron-fortified foods as a viable vehicle to contribute to the intake of bioavailable iron, since several international public health interventions have already demonstrated the effectiveness of this type of action. Finally, national public campaigns are needed to inform the Brazilian parents and caregivers regarding the importance of adequate iron consumption in children and to bring awareness regarding the food practices that can help enhance the absorption of iron, taking in consideration the current diets of the Brazilian children.

## Data availability statement

The raw data supporting the conclusions of this article will be made available by the authors, without undue reservation.

## Ethics statement

The survey protocol and data collection instruments were approved by the institutional review boards of RTI International, Faculdade de Ciências Farmacêuticas from University of São Paulo and the National Commission for Research Ethics (Comissão Nacional de Ética em Pesquisa). Written informed consent to participate in this study was provided by the participants' legal guardian/next of kin.

## Author contributions

CN-d-A: substantial contributions to the conception or design of the work, drafting the work or revising it critically for important intellectual content, final approval of the version to be published, figures, study design, data collection, data interpretation, and data analyses. DP: substantial contributions to the conception or design of the work, or the acquisition, analysis, or interpretation of data for the work, drafting the work or revising it critically for important intellectual content, final approval of the version to be published, and data analyses. EM: substantial contributions to the conception of the work, final approval of the version to be published, and data interpretation. FU: substantial contributions to the conception or design of the work, or interpretation of data for the work, drafting the work, final approval of the version to be published, data collection, and data interpretation. VC: substantial contributions to the conception or design of the work, or the acquisition, analysis, or interpretation of data for the work, drafting the work or revising it critically for important intellectual content, final approval of the version to be published, literature search, data analysis, and data interpretation. All authors contributed to the article and approved the submitted version.
